# Micro- and Nanoplastics as Disruptors of the Endocrine System—A Review of the Threats and Consequences Associated with Plastic Exposure

**DOI:** 10.3390/ijms26136156

**Published:** 2025-06-26

**Authors:** Hanna J. Tyc, Karolina Kłodnicka, Barbara Teresińska, Robert Karpiński, Jolanta Flieger, Jacek Baj

**Affiliations:** 1 Department of Correct, Clinical, and Imaging Anatomy, Chair of Fundamental Sciences, Medical University of Lublin, Jaczewskiego 4, 20-090 Lublin, Poland; 2 Department of Forensic Medicine, Medical University of Lublin, Jaczewskiego 8b, 20-090 Lublin, Polandb.teresinska2@gmail.com (B.T.); 3 Department of Machine Design and Mechatronics, Faculty of Mechanical Engineering, Lublin University of Technology, ul. Nadbystrzycka 36, 20-618 Lublin, Poland; 4 Institute of Medical Sciences, The John Paul II Catholic University of Lublin, ul. Konstantynów 1H, 20-708 Lublin, Poland; 5 Department of Analytical Chemistry, Medical University of Lublin, Chodźki 4A, 20-093 Lublin, Poland; jolanta.flieger@umlub.pl

**Keywords:** endocrine-disrupting chemicals (EDCs), endocrine system, microplastics, nanoplastics

## Abstract

Plastic overconsumption has emerged as a major environmental pollutant, with degraded micro- and nanoplastic (MNP) particles being consumed by a vast variety of species. MNPs, particles < 5 mm, contain endocrine-disrupting chemicals (EDCs), which can bind to hormone receptors and disrupt the proper endocrinological function of a variety of organs. This review explores the toxicological impact of MNPs on the hypothalamus, pituitary gland, thyroid, pineal body, ovaries, and testes, as well as the effects of the endocrinological regulatory axes, including the hypothalamic–pituitary–gonadal (HPG), hypothalamic–pituitary–thyroid (HPT), and hypothalamic–pituitary–adrenal (HPA) axes. The disruption of these hormonal feedback systems leads to reproductive dysfunction, neurotoxicity, cytotoxicity, immunotoxicity, and metabolic disorders. The gonads are particularly susceptible, with studies demonstrating oxidative stress, cellular apoptosis, and infertility due to MNP exposure. Given the widespread presence of MNPs and their impact on human health, further research is critical to understand their long-term effects and develop strategies to reduce exposure.

## 1. Introduction

In the past decades, plastic creation and use have exponentially grown, as has the human consumption of MNPs, with approximately > 50,000 particles ingested per year [1]. Nanoplastics (NPs) are classified as plastic particles with a size < 1 μm, and microplastics (MPs) are plastics with a size < 5 mm [2,3]. Plastics contain many endocrine-disrupting chemicals (EDCs), which can leach into liquids and cause dysregulation of the endocrine organs, leading to the disruption of reproductive function, neurotoxicity, immunotoxicity, cytotoxicity, and developmental abnormalities [2,3,4,5,6]. EDCs exhibit similar properties as hormones and can act at low concentration levels [3]. Some common EDCs include phthalates, bisphenol A (BPA), polybrominated diphenyl ethers (PBDEs), tributyltin (TBT), polychlorinated biphenyls (PCBs), chromium, lead, and mercury [2,3,7,8]. The human body may be exposed to MNPs through inhalation, ingestion, and dermal contact [9,10,11,12,13]. The accumulation of MNPs has been found in many organ systems and samples of blood, breast milk, sputum, feces, and urine [10,14,15,16]. These MNPs can absorb and transport harmful chemicals, such as EDCs, and have a toxic effect on the tissue and organs in which they accumulate. Toxicity is size- and dose-dependent, with smaller particles having a greater surface area and better absorptive capacity, releasing more toxic chemicals, and a larger dose is also able to carry more chemicals [2,9]. MNPs have been found in nearly all components of the natural environment, including surface and deep ocean waters, sediments, freshwater bodies, soils, and even the air [17,18]. Their presence has also been proven in Arctic ice and inaccessible mountainous areas, demonstrating their ability to travel large distances by wind and water currents [19]. MNPs pose significant threats to wildlife, particularly aquatic creatures. Marine creatures frequently confuse MPs for food, resulting in gastrointestinal obstructions, starvation, and diminished reproductive potential [20]. Filter-feeding species such as mussels and plankton can accumulate nanoplastics, which can disrupt biological functioning and provoke inflammatory responses [21]. Similarly, terrestrial animals exposed to tainted water or food may suffer from oxidative stress or hormonal disturbances [22,23]. New research reveals that plants can absorb nanoplastics through their roots, potentially impairing root growth, nutrient uptake, and photosynthesis, compromising crop productivity and ecosystem balance (Table 1) [24].

The aim of this review article is to provide a comprehensive analysis of the toxicity of MNPs and their associated EDCs in relation to dysfunction of the endocrine system in humans. This work outlines the current knowledge regarding the effects of MNPs on key hormonal regulatory axes (HPG, HPT, HPA) and individual endocrine glands, including the hypothalamus, pituitary gland, thyroid gland, adrenal glands, ovaries, and testes. This article seeks to highlight the potential health consequences resulting from MNP exposure and to identify critical research gaps relevant for future epidemiological and toxicological studies.

## 2. Routes of Exposure to Micro- and Nanoplastics in Humans

**Figure 1:**
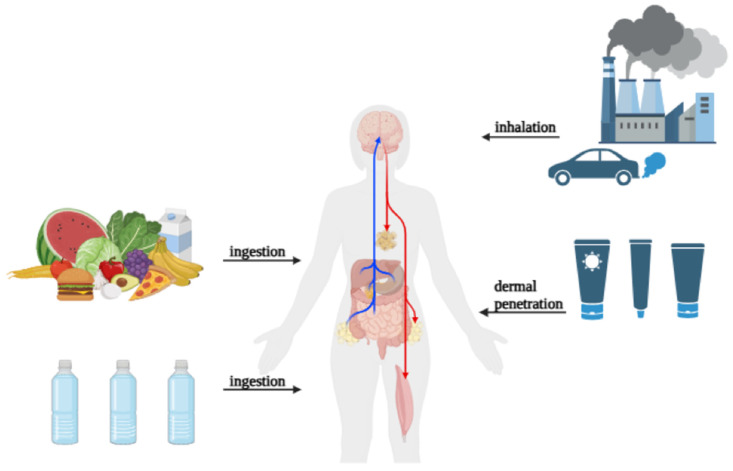
Potential routes of micro-/nanoplastic exposure.

Humans can be exposed to MNPs in three ways: ingestion, inhalation, and dermal contact. These pathways of exposure are interrelated and contribute to the total amount of plastic particles in the human body. MPs are thought to most commonly be ingested. The principal sources are contaminated food and drinkable water. MPs have been found in a variety of products, including seafood, salt, sugar, tea bags, and milk [25]. According to many studies, Europeans consume between 39,000 and 52,000 MP particles per year through food consumption, with an extra 11,000 particles coming from eating shellfish [26]. Take-out food containers comprising common polymer materials such as PP, PS, PE, and PET are extensively used and have been discovered to contain MPs [27]. Individuals who order takeout food four to seven times a week may consume between 12 and 203 MP particles through containers [28]. Furthermore, studies show that silicone rubber baby teats deteriorate after steam sterilization, releasing MP particles into the environment [29]. It is estimated that the total quantity of MP particles entering a baby’s body throughout one year of typical bottle feeding is around 0.66 million [30]. Another major source of MP exposure is inhalation. Airborne MPs are made up of PE, PS, and PET particles and fibers, ranging in size from 10 to 8000 µm. The majority of MPs in the atmosphere (84%) come from automobile activity. MP fiber concentrations in outdoor and indoor air have been found to vary according to studies [31]. In Paris, the median concentration of MP fibers in outdoor air is 5.4 fibers/m^3^, whereas interior air has 0.9 fibers/m^3^ [32]. When oral intake and inhalation are combined, annual MP consumption is predicted to be between 74,000 and 121,000 particles [33]. Infants and children have been found to spend more time indoors, possibly contributing to increased susceptibility as a population group. Although the concentration of MPs in indoor air below 1 m height has not been well documented, children are much more likely to inhale and ingest dust particles. Indoor dust has been found to have a high concentration of MPs, ranging from 38 to 1.2 × 10^5^ μg/g to <0.11 to 1700 μg/g in samples from 12 countries. Further, while adult estimates of annual MP consumption range from 4.0 × 10^2^–2.5 × 10^4^ particles, it is estimated that infants and young children consume 7.2 × 10^2^–4.5 × 10^4^ particles, showing a 1.8× larger consumption in the child population. Continually, during inspection of MPs in human feces, concentrations were found to be higher in infantile fecal matter than in adults, suggesting a higher exposure rate [34]. Dermal contact with MPs is an understudied but conceivable mode of exposure. MPs are generally thought to not pass through the skin barrier [35]. However, they can enhance exposure risk by accumulating on the skin. The usage of MP-containing consumer items, such as face creams and cleaners, increases the risk of PE exposure [36,37]. MPs can be generated by protective mobile phone cases during use, which are then transferred to human hands [38]. Some common plastic additives, such as brominated flame retardants, BPAs, triclosan, and phthalates, can be absorbed through cutaneous contact with MPs [39]. These chemicals are known to affect endocrine function and may increase health risks (Figure 1).

## 3. Accumulation of Micro- and Nanoplastics Within the Endocrine System

### 3.1. Hypothalamus

The hypothalamus controls the release of hormones by the pituitary gland, as well as water balance, sleep, temperature, appetite, and blood pressure. The hypothalamus produces and secretes thyrotropin-releasing hormone (TRH), gonadotropin-releasing hormone (GnRH), growth-hormone-releasing hormone (GHRH), corticotropin-releasing hormone (CRH), somatostatin, and dopamine, which travel to the anterior pituitary, and it also creates oxytocin and antidiuretic hormone (ADH), which are stored in the posterior pituitary. MPs act through their EDCs to disrupt the feedback of the HPT and the HPG axes [2,40,41]. This action mainly occurs due to PBDEs, an EDC typically found in polyurethane foam [2]. Dysregulation of these axes can also be caused by phthalates, TBT, BPA, PCBs, mercury, and chromium. The effects of these EDCs are not only through disruption of feedback mechanisms but also due to a decrease in hypothalamic neurons, decreased hypothalamic weight, oxidative stress in the hypothalamus, astrocyte activation leading to astrocyte-dependent hypothalamic inflammation, changes in hypothalamic neuropeptides, and distortion of gene expression [2,40]. In a study observing the effects of PS-NPs on zebrafish, behavioral changes and impaired aggressiveness occurred due to a decrease in the neuropeptides vasopressin (ADH) and oxytocin. Both hormones are directly controlled and created by the hypothalamus, demonstrating the dysregulation of this gland [42,43]. The dysregulation of the hypothalamus causes harmful disorders of many endocrine organs due to its major role in its axes.

### 3.2. Pituitary Gland

**Figure 2:**
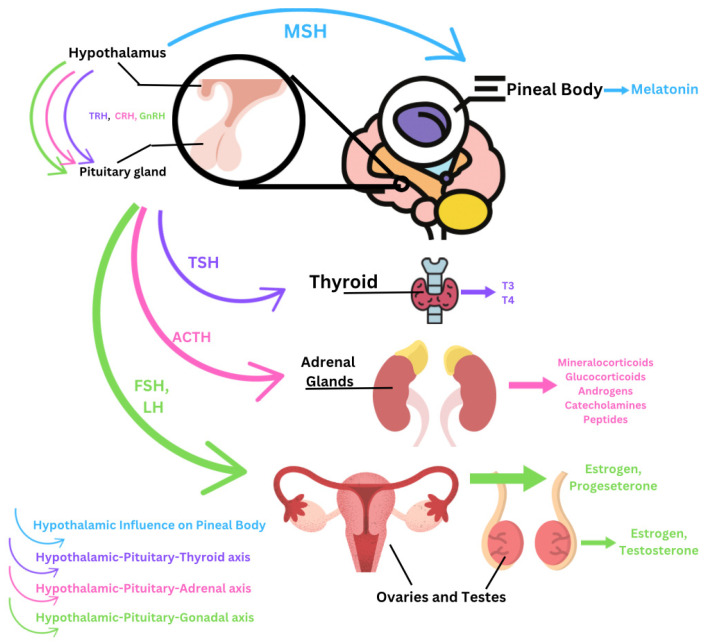
Hormonal axes and their main hormones.

The pituitary gland is divided into two segments, the anterior and posterior pituitary. The anterior pituitary gland produces and secretes adrenocorticotropic hormone (ACTH), thyroid-stimulating hormone (TSH), luteinizing hormone (LH), follicle-stimulating hormone (FSH), prolactin (PRL), growth hormone (GH), and melanocyte-stimulating hormone (MSH). The posterior pituitary stores and secretes ADH and oxytocin. Through the action of these hormones, the pituitary acts on several axes, including the HPT, HPG, and the HPA axes, to regulate growth, reproduction and sexual function, and metabolism through the thyroid, adrenal glands, and gonads (ovary and testis) [2,10,42]. EDCs mainly act to dysregulate the pituitary gland indirectly by its actions on the hypothalamus. The levels of the hormones GnRH, FSH, and LH are typically reduced or inhibited. This causes extreme disruption of the reproductive system, leading to decreased fertility or infertility and possible carcinogenic effects on the uterus [43]. Further, EDCs can lead to the formation of a prolactinoma, a benign pituitary tumor, which can cause abnormal stimulation of the release of PRL and TSH [10,44]. Additionally, chromium found in certain plastics can cause oxidative stress and increased superoxide dismutase activity in the anterior pituitary (Figure 2) [2].

Through actions on the pituitary gland, EDCs also indirectly increase stimulation of the adrenal gland and dysregulate corticosterone and aldosterone levels [2,42]. EDCs increase the size of the adrenals, increasing the mass of the zona fascicula, causing an increase in glucocorticoid secretion. The zona medulla demonstrates impaired function, decreasing levels of secreted catecholamines, norepinephrine, and epinephrine. EDCs, specifically TBT, cause an increased accumulation of lipids in the intracellular storage of the adrenal cells, ultimately increasing cholesterol levels. The effects on the adrenal glands directly or indirectly through the pituitary gland ultimately leads to hypertension and negative metabolic effects [2].

### 3.3. Thyroid Gland

The thyroid gland plays a critical role in metabolism, development, and thermoregulation through the production of thyroxine (T4) and triiodothyronine (T3). Dysregulation of TSH can cause detrimental effects on the thyroid gland. Long-term exposure to MNPs impairs the growth, development, metabolism, and reproduction of the thyroid gland. PBDEs, BPA, phthalates, and organotin may act directly on the thyroid gland and are thus termed thyroid-disrupting chemicals (TDCs). During childhood exposure, these chemicals cause a reduction in thyroid weight, hyperactivity, and developmental abnormalities. They may interfere with T3 and T4, respectively, by disrupting the HPT axis [1,2,45]. They may inhibit T3 receptor binding and impair transcriptional activity mediated by thyroid hormone receptors [1,2]. They may also interfere with gene expression and alter the gene expression of the TSH subunit, such as deiodinase type 2 (deio2) and NK2 homeobox 1 (nkx2.1) [2]. Furthermore, certain EDCs can inhibit the sodium–iodine symporter (NIS), which is required for iodine uptake in the thyroid gland [46]. These chemicals reduce NIS expression or function, impairing iodide transport and, as a result, thyroid hormone production [47]. Moreover, several plastic-associated chemicals may stimulate hepatic glucuronidation enzymes, accelerating T4 metabolism and lowering circulating hormone levels, resulting in compensatory TSH rise and possible goiter formation [48,49]. Epidemiological research suggests that long-term exposure to MNPs may contribute to subclinical thyroid disease (SCTD), especially in sensitive populations like children and pregnant women [10,34]. Penetration and transversion of the intestinal, respiratory, and placental barriers and the ability to disrupt these barriers enhance susceptibility during pregnancy. Barrier penetration and placental MNP accumulation increases maternal–fetal transfer of EDCs, potentially affecting thyroid hormone homeostasis in both mother and fetus. Further, placental inflammation and oxidative stress may perturb thyroid hormone synthesis or peripheral metabolism, contributing to subclinical hypothyroidism [10,34]. Additionally, fetal and early postnatal exposure to MNPs can disrupt developmental programming, especially as the endocrine and immune system are still forming [10]. In young children, biological barriers and metabolic clearance systems are underdeveloped, leading to greater tissue accumulation of MNPs and their associated EDCs. This is especially seen in organ systems characterized by frequent cell division or hormonal regulation, such as the thyroid [34]. SCTD has been connected to neurodevelopmental abnormalities, cardiovascular risk, metabolic dysregulation, and a decrease in fertility [50]. Ultimately, prolonged MNP exposure causes thyroid hormone imbalance, altered gene expression, and possibly long-term systemic health effects [51].

### 3.4. Parathyroids

The parathyroid glands are responsible for regulating calcium and phosphate homeostasis by secreting parathyroid hormone (PTH) [52]. PTH raises blood calcium levels via boosting calcium release from bones [53]. It improves renal calcium reabsorption and activates vitamin D in the kidneys [54]. Exposure to MNPs and EDCs can impair the function of these glands. According to research, EDCs like BPA and phthalates can disrupt calcium homeostasis by disrupting the parathyroid glands [2]. These substances can dysregulate PTH secretion either directly on parathyroid cells or by influencing calcium-sensing receptors (CaSRs) and other PTH-related regulatory processes [55,56]. Some research implies that BPA exposure increases PTH levels, while others claim that it reduces PTH secretion due to altered calcium signaling [57]. Furthermore, MNPs and EDCs can have an indirect effect on calcium metabolism by influencing renal function, where PTH has the greatest impact [2]. For example, BPA has been related to reduced renal function, which may result in improper calcium reabsorption and worsen calcium imbalances in the body [58]. These abnormalities in young and developing organisms may lead to the development of bone disorders such as osteopenia and osteomalacia. Chronic exposure to these substances may cause an imbalance in calcium and phosphate metabolism, raising the risk of bone fragility, especially in developing children or those with impaired kidney function [59,60]. However, much of the data on the effects of MNPs and EDCs on parathyroid function is preliminary, and further studies are needed to fully understand the processes at work and the long-term health implications.

### 3.5. Adrenal Glands

The adrenal glands, located above the kidneys, regulate stress reactions, metabolism, immunological function, and fluid balance. They are made up of two parts: the adrenal cortex, which secretes corticosteroids (cortisol and aldosterone), and the adrenal medulla, which generates catecholamines (adrenaline and noradrenaline). These hormones play a key role in stress response, electrolyte balance, and metabolic regulation [61]. Exposure to MNPs and EDCs can have a major impact on adrenal function, both directly and indirectly via the HPA axis. MNPs and EDCs have been demonstrated to disrupt the HPA axis, resulting in an imbalance in cortisol levels, either by overstimulating or suppressing production [2]. Elevated cortisol levels, known as hypercortisolism, can cause a variety of metabolic problems, including insulin resistance, obesity, hypertension, and an increased susceptibility to infection [62]. In addition to influencing cortisol release, MNPs and EDCs can influence catecholamine synthesis in the adrenal medulla. Some studies have found that environmental contaminants cause lower catecholamine secretion, which may impede the body’s capacity to mount an effective stress response [63,64]. Chronic exposure to these substances may have long-term consequences on stress resilience, possibly contributing to anxiety, depression, and other mood disorders [65]. Furthermore, certain MNPs and EDCs might induce structural damage to the adrenal glands. For example, certain substances may cause lipid buildup in the adrenal cortex, limiting corticosteroid synthesis and resulting in metabolic dysregulation [66]. Long-term exposure may cause adrenal atrophy or hyperplasia, affecting blood pressure and fluid balance, which is commonly seen with dysregulated aldosterone levels. The health ramifications of impaired adrenal function caused by plastic-derived toxins are far-reaching [67]. In addition to the metabolic impacts, development of cardiovascular problems, hormonal imbalances, and weakened immune systems may occur [13]. While much of the information is preliminary, the expanding corpus of the research highlights the importance of future exploration into how MNPs and EDCs affect adrenal function and the broader endocrine system.

### 3.6. Pineal Body

The pineal body functions to control the circadian cycle by stimulation from light/darkness in the environment [68]. In response to darkness, the pineal body produces and secretes melatonin. High doses of NPs have been seen to dysregulate the circadian rhythm by impairing the pineal gland and its production and secretion of melatonin [42]. This was demonstrated in zebrafish, where chronic exposure to MPs significantly affected the circadian rhythm locomotion activity, specifically during the light cycle, demonstrating reduced locomotion and abnormal movement orientation [42]. Moreover, in zebrafish, melatonin has been found to ameliorate the detrimental neurodevelopmental effects of NP-PS and alleviate damage from MPs on the intestines [69,70]. Melatonin also has antioxidant, anti-inflammatory, and anti-apoptotic effects and has demonstrated protective effects on mitochondria. The accumulation of MPs may upregulate mitochondrial ROS production and secretion, causing oxidative stress and damage to many tissues. Melatonin has been found to have a protective effect, mitigating PS-NP-induced mitochondrial dysfunction. However, the impairment of the regulatory cycle of melatonin due to the accumulation of MPs in the pineal gland prevents this physiological protective effect of melatonin [71].

### 3.7. Ovaries

The ovaries have both reproductive and endocrinological functions, responsible for producing oocytes for fertilization and estrogen and progesterone to regulate the menstrual cycle and produce female characteristics. The hormonal expression of the ovaries is controlled by FSH and LH secreted from the anterior pituitary, as mentioned in Section 3.2. Oral exposure to MPs leads to accumulation within the uterine tissue and the ovaries [2,10,72]. Ovaries have been found to have a reduced weight after exposure to MPs and decreased cytoskeletal proteins [10]. As demonstrated in mice studies, ovaries undergo oxidative stress, increased MDA levels, granulosa cell apoptosis, ovary fibrosis, and pyroptosis when exposed to MPs [2,44,73]. Apoptosis and oxidative stress in the ovaries lead to downregulation of Bcl-2 and upregulation of Bax in granulosa cells, which adversely affects female fertility and impairs ovarian cells essential for oocyte development [73,74]. Further, a direct decrease in pregnancies and increased mortality has been observed [2]. In marine medaka, exposure to MPs led to a reduced number of spawning follicles and an increase in early vitellogenic oocytes [43,75]. Follicular atresia, low follicle growth, and corpus luteum have been seen in mice, rats, and rabbits [2]. Disruption of follicular growth may be caused by cyp1a1 downregulation due to the accumulation of MPs [42]. In mice, due to the presence of MPs, a decreased thickness of the granulosa layer in secondary follicles and a reduced number of growing follicles were observed. Likewise, a substantial accumulation of ovarian collagen, fibronectin, and fibrotic processes in the ovaries and granulosa cells was detected. This mainly occurred due to increased levels of reactive oxygen species (ROS) and malondialdehyde (MDA), while antioxidant activity was simultaneously decreased, including superoxide dismutase (SOD), catalase (CAT), and glutathione peroxidase (GPx) [44].

In females, an increase in circulating LH, FSH, and T and a decrease in estradiol and anti-Mullerian hormone (AMH) are seen due to disruption of the HPG axis and defective ovarian steroidogenesis. These changes in hormone balances dysregulate the typical menstrual cycle and bleeding time and may lead to masculinization [2,10,44,73]. In female seahorses, androgen production was increased, which led to atresia of ovarian follicles, zona pellucida breakdown, yolk liquefaction, and hypertrophy of the granulosa cells in the ovaries [76]. Additionally, in fish, MPs have been shown to delay the development of ovaries due to defective estrogen production [43]. In rodents, measurable changes to the estrous cycle duration decreased ovarian reserve, and lower implantation rates occurred due to MP exposure, mostly due to oxidative stress. Evidence of oxidative stress markers such as MDA has been seen after exposure to MPs, supporting this theory [10]. Human granulosa cells in vitro also demonstrate increased evidence of lipid peroxidation after MP exposure due to decreased levels of superoxide dismutase (SOD2) and glutathione (GSH) antioxidant systems and decreased cell viability. These changes may also promote excess fibroblast proliferation and fibrosis of the ovarian and uterine tissue [10].

In the presence of MPs, bovine oocytes fail to mature, demonstrating significant proteomic alteration, in part by increasing apoptotic gene expression [10,44]. In female mice, BPA impairs cytoskeletal dynamics in the oocyte, induces oxidative stress, and may increase DNA damage and epigenetic alteration in oocytes [2,44,77]. While lambs exposed to BPA exhibit reduced ovarian follicular reserves, a smaller population of primordial follicles, an increase in antral atretic follicles, a higher prevalence of follicles containing multiple oocytes, and lower ovarian weights are noted [44]. BPA has also been observed to directly decrease the maturation of oocytes. It disrupts oocyte-secreted proteins like GDF9 and CX37, damages gap junctional intercellular communication in cumulus–oocyte complexes (COCs), and impairs the transition of oocytes from prophase I to metaphase II (Table 2) [44].

### 3.8. Testes

Testes produce male gametes and testosterone and play a role in the HPG axis to regulate reproductive function. MPs have been found to enter and situate in the male testes, leading to changes in reproductive and physiological function of the organ, mainly through oxidative stress but also through testicular inflammation, activation of prooxidant mediators, cell death, and inhibition of antioxidant mechanisms [2,41,43,44,78]. Abnormal spermiogenesis and reproductive functions of the testes have led to recent interest in medical studies based on toxicity of MPs and, specifically, their effects on the reproductive organs. MPs have been observed to accumulate in mice testes and testicular tissues, including germ cells, Sertoli cells, and Leydig cells [2]. In zebrafish, PS-NPs have been witnessed to accumulate in the testes, affecting reproductive health [9,73]. MNP accumulation causes a reduction in testosterone production, directly affecting the quality, number, and vitality of sperm, as well as causing demasculinization [2,41,43]. In the germinal tubules, H&E staining showed multinucleated gonadotrophic cells and disorganized spermatogonia [41]. This may in part be caused indirectly by testicular inflammation due to the accumulated MPs. In a mouse study, exposure to MPs during developmental stages of the testes caused morphological changes in individual sperm, including loss of sperm acrosome, small-headed sperm (cephalic), headless sperm (acephalic), and tailless sperm [2,44,79]. An increase in SOD and MDA in the testes due to exposure to MPs suggests the involvement of oxidative pathways in the disruption of testicular function, similar to what is seen in ovaries (Section 3.7) [44]. Reproductive function is heavily reliant on the HPG axis, which, as mentioned in Section 3.2, is heavily dysregulated due to the EDCs found in plastic. Consequently, due to the dysregulation of the HPG axis and antiandrogenic effects of MPs, adverse steroidogenic enzyme activity is also seen, which may in part be responsible for defective spermatozoa [2].

Apart from reduced sperm quality, the rearrangement of the spermatids in the seminiferous tubules creates a decreased sperm density and reduced spermatid number [2,9,44]. Degeneration of the seminiferous tubules is marked by disorganized, shrunken tubules and irregular, buckled basement membranes [9,41,43]. Disruption of the blood–testis barrier (BTB) has been noted and may be caused by the activation of the p38 MAPK-nuclear factor erythroid-2-related factor 2 (Nrf2) pathway. This pathway may also lead to vas deferens damage as well as spermatogenic cell apoptosis [41]. Due to MP presence in the testes, NF-kB was upregulated, initiating the apoptosis of testicular cells in male marine medaka [43]. MP exposure leads to spermatogenic cells showing pyknosis, nuclear rupture, and detachment [43]. Likewise, in mice studies, an altered sperm phenotype has been observed. Germ cells in the seminiferous tubules have also been observed to shrink due to the presence of MPs [1]. Testicular weight is also decreased, and a decreased viability of testicular cells is observed. Overall, significant pathomorphological changes occurred in rodents’ testes due to the accumulation of MNPs [2,9,44]. It is important to note that changes in semen quality are directly related to the exposure dose of the MPs [41].

In Sertoli cells, MPs have been found to cross the BTB, dysregulate spermatogenesis, and decrease T secretion [2,43,44]. MNPs and their associated EDCs can disrupt the tight junctional proteins of the BTB and cause a reduction in protein expressions such as claudin 11, N-cadherin, connexin, and occludin [2]. These proteins are responsible for the formation of specialized tight junctions via the connection of Sertoli cells, shielding developing germ cells from harmful substances and immune system attacks. Disruption of tight junction proteins, therefore, increases BTB permeability [2]. The impacted BTB integrity due to MPs can also in part be due to an ROS-induced change in the balance of mTORC1 and mTORC2 signaling. This alters the expression of actin cytoskeleton components, causing structural damage and inflammation-mediated opening of the BTB. Further, disruption of this signaling pathway may directly cause dysfunction of spermatogenesis [43]. In male seahorses, TBT diminished spermatogenesis and sperm motility through suppression of cyclic AMP, as well as androgen synthesis, and caused degeneration of the testes [76]. MPs work to disrupt spermatogenesis by causing ablation and irregular positioning of spermatogenic cells. However, the primary mechanism for reproductive impairment is due to sperm DNA fragmentation and testicular transcriptomic alterations. In males, dysregulated sperm DNA methylation has also been seen [2,41,73].

In both males and females, an inflammatory effect of MPs is seen. The accumulation of MPs causes an increase in the abundance of inflammatory factors and ROS such as TNF-alpha, interleukin-1beta, IL-6, IL-8, CAT, peroxidase, GSH, glutathione peroxidase or glutathione-S-transferase, and apoptotic factor caspase-3 [43,44]. It is suggested the rise in ROS and subsequent MAPK signaling may cause DNA damage in the testicular tissue beyond the damage that occurs to DNA due to oxidative stress alone [43]. The increase in ROS may be due to upregulated mitochondrial activity in response to the accumulation of MPs resulting in increased ROS generation [43,80]. In male mice exposed to PS-MPs, the concentration of FSH, LH, and T4 is decreased, and estradiol levels are increased; this is the complete opposite of what occurs in females (Section 3.7) [44]. Comprehensively, the exposure and accumulation of MNPs in the testes diminish and inhibit the reproductive function of the testes through hormonal and pathomorphological alterations (Table 3).

## 4. Current Challenges and Limitations and Research Directions for Future Studies

Although there has been increasing knowledge on how MNPs affect conventional endocrine organs including the ovaries, testes, and thyroid, information about other organs, especially the parathyroid and adrenal glands, is still in very early stages of research. These knowledge gaps indicate the research infancy of MNPs and their influence on the endocrine system and emphasize the necessity for cross-disciplinary fundamental studies, including analysis of the transcriptome and proteome, organoid models, and in vivo exposure across different species. Upcoming studies should concentrate on the unexplored endocrine-disrupting impact of MNPs on the calcium-regulatory (e.g., parathyroid) and stress-related (e.g., HPA axis) systems. Furthermore, there is a lack of research about the potential risk assessments of MNPs. Subsequent manuscripts should concentrate on developing knowledge about risk evaluation as well. Future directions may involve extending traditional toxicological methods to account for the diverse circumstances of human exposure, including low-dose, multi-route, chronic exposure, and mixtures with other contaminants. Future studies ought to focus on measuring MNP levels in human blood, urine, placenta, and other tissues through biomonitoring research; modeling dose–response relationships based on longitudinal exposure data and actual concentrations; toxicokinetic models to evaluate MNP intake, distribution, metabolism, and elimination in humans; and utilizing data omics to identify early biomarkers of endocrine toxicity. Moreover, there is a necessity for quantitative human health risk assessment and experimental toxicology to be connected by population-based epidemiological investigations that link the impacts of MNPs on endocrine diseases.

The field of study about harmful effects on the endocrine system has a number of fundamental limitations, despite its quickly expanding interest. The limitations include animal-to-human extrapolation, limited by species diversity. Theoretical conclusions become challenging when exposure to MNPs and chemical compounds is combined and there is a lack of long-term human data. We suggest a coordinated, multidisciplinary strategy for filling these gaps. This would entail global research collaborations to exchange data and standardize methods, the establishment of high-throughput screening procedures for MNPs and their combined ability to alter hormones, and the adoption of systems of biology techniques to simulate the endocrine effects across multiple organs. Additionally, initiating biomonitoring studies that include vulnerable groups, such as pregnant women and infants, will enable science to progress from descriptive toxicology to prognostic risk modeling and public health policy recommendations.

## 5. Conclusions

The vast plethora of plastics and plastic degradation are creating a so-called plastic epidemic, with consumption of MNPs at an all-time high. The immense amount of MNPs accumulated in the human body greatly disrupts the endocrinological system, dysregulating reproductive function, growth and development, metabolism, and neurological and immune function. Endocrine organs such as the thyroid, parathyroid, and adrenal glands are especially affected by hormone imbalance, receptor interference, altered gene expression, and disrupted calcium and stress hormone production. With growing evidence of plastic consumption and its negative effects on the human body, especially in vulnerable populations like children and pregnant women, it is important to limit the mass production of plastics, especially the production of EDC additives. Further research is essential to explore the long-term effects and determine the exact mechanism of toxicity of plastics. Given the increasing evidence of endocrine and systemic toxicity associated with MNP, often compounded by co-exposure to metal contaminants, future research should prioritize elucidating molecular and epigenetic mechanisms of action, identifying early biomarkers of toxicity, and translating experimental findings into human health risk assessments to inform regulatory strategies.

## Figures and Tables

**Table 1:** Types of plastic in the environment. This table presents the main types of plastic found in the environment in the last few years. The table also presents typical uses and their degradation characteristics.

Type of Plastic	Common Uses	Environmental Characteristics
Polyethylene (PE)	Plastic bags, bottles	Very resistant to degradation, widespread in oceans and soils
Polypropylene (PP)	Food containers, straws, bottle caps	Floats in water, slowly degrades under UV exposure
Polystyrene (PS)	Disposable cutlery, insulation materials	Brittle material, which breaks into small particles
Polyvinyl chloride (PVC)	Pipes, medical devices, flooring	Releases harmful additives
Polyethylene terephthalate (PET)	Beverage bottles, clothing fibers	Recyclable but persistent, common in marine environments
Polycarbonate (PC)	Electronic devices, eyewear lenses, water bottles	Can leach BPA and forms small, durable fragments

**Table 2:** Effects of MNPs on the ovaries.

Effects on Ovaries	Description
Reproductive impact	Reduced ovarian weight, fewer follicles, decreased fertility, impaired oocyte maturation
Oxidative stress	Increased ROS and MDA, decreased antioxidant activity (SOD, CAT, GPx), granulosa cell apoptosis
Fibrosis	Accumulation of collagen and fibronectin in ovaries, ovarian and uterine tissue fibrosis
Hormonal imbalance	Increased LH, FSH, and T, decreased estradiol and AMH, disrupted menstrual cycle, potential masculinization
Follicular development	Delayed follicle growth, increased atresia, zona pellucida breakdown in various species

**Table 3:** Effects of MNPs on testes.

Effects on Testes	Details
Reproductive function	Reduced sperm quality, abnormal spermatogenesis, decreased sperm count, impaired motility.
Oxidative stress	Increased ROS and MDA, decreased antioxidant activity (SOD, CAT, GPx), testicular inflammation, cell death.
Hormonal disruption	Reduced testosterone production, dysregulated HPG axis, antiandrogenic effects, altered sperm phenotype
Structural damage	Seminiferous tubule degeneration, disrupted BTB, shrunken germ cells, reduced testicular weight.
Inflammatory response	Increased TNF-alpha, IL-1beta, and IL-6, activation of apoptotic factors (caspase-3), DNA damage in testes

## Abbreviations

ACTH	adrenocorticotropic hormone
ADH	antidiuretic hormone
AMH	anti-Mullerian hormone
BPA	bisphenol A
BTB	blood–testes barrier
CaSRs	calcium-sensing receptors
CAT	catalase
COCs	cumulus–oocyte complexes
CRH	corticotropin-releasing hormone
deio2	deiodinase type 2
EDCs	endocrine-disrupting chemicals
FSH	follicle-stimulating hormone
GH	growth hormone
GHRH	growth-hormone-releasing hormone
GnRH	gonadotropin-releasing hormone
GPx	glutathione peroxidase
GSH	glutathione
HPG	hypothalamic–pituitary–gonadal
HPA	hypothalamic–pituitary–adrenal
HPT	hypothalamic–pituitary–thyroid
LH	luteinizing hormone
MDA	malondialdehyde
MNPs	micro- and nanoplastics
MP	microplastic
MPs	microplastics
MSH	melanocyte-stimulating hormone
NIS	sodium–iodine symporter
NP	PS nanoplastic–polystyrene
NPs	nanoplastics
Nrf2	nuclear factor erythroid-2-related factor 2
PBDEs	polybrominated diphenyl ethers
PCBs	polychlorinated biphenyls
PE	polyethylene
PP	polypropylene
PRL	prolactin
PS	polystyrene
PTH	parathyroid hormone
ROS	reactive oxygen species
SCTD	subclinical thyroid disease
SOD	superoxide dismutase
SOD2	superoxide dismutase
T3	triiodothyronine
T4	thyroxine
TBT	tributyltin
TDCs	thyroid-disrupting chemicals
TRH	thyrotropin-releasing hormone
TSH	thyroid-stimulating hormone
nkx2.1	NK2 homeobox 1
